# Osteochondritis Dissecans of the Capitellum: A Case Report of Successful Arthroscopic Treatment

**DOI:** 10.1155/2017/5086542

**Published:** 2017-03-26

**Authors:** J. Ribeiras Cabral, R. Henriques, J. Arvela Matoso, S. Martins, M. Sarmento

**Affiliations:** Orthopedic Surgery Department, Hospital de Santa Maria, Av. Prof. Egas Moniz, 1649-035 Lisboa, Portugal

## Abstract

*Introduction.* Osteochondritis dissecans (OCD) of the capitellum is a localized disorder of the subchondral bone, in a region with limited healing capacity. Although its aetiology is still unknown, it has been associated with repetitive microtrauma. The natural history of this disease involves the evolution for degenerative joint disease in approximately half of the patients, with early identification and treatment being critical to optimizing the outcome.* Case Presentation.* We present a rare case in our practice, illustrating a capitellar OCD in a fifteen-year-old White male without an identified cause of repetitive microtrauma.* Conclusion.* In this case prompt diagnosis and arthroscopic-assisted treatment led to a successful result.

## 1. Introduction

Osteochondritis dissecans (OCD) of the capitellum is a localized disorder of the subchondral bone resulting in fragmentation of the articular surface and underlying bone [1–3].

Although its aetiology is still unknown, there is strong suspicion of its relation with repetitive microtrauma with a valgus stress in a relatively poor vascularized subchondral bone [3, 4].

This theory is supported by the fact that OCD of the capitellum typically affects the young athlete involved in high-demand, repetitive, overhead, or weightbearing activities, such as baseball and gymnastics [4, 5]. In fact, the studies attempting to find the prevalence of OCD of the capitellum were performed in the subpopulation of young male baseball players, where it peaks between 2,1 and 3,4% [6, 7].

The capitellar lesion is focal and has limited capacity for healing [3]. The repetitive microtrauma weakens the subchondral support of the articular surface and, if left unchecked, results in lesion progression with flattening, fragmentation, and formation of loose bodies [4, 8].

Long-term results show the evolution for degenerative joint disease and maintenance of elbow symptoms in approximately half of the patients [9, 10].

We present a rare case in our practice, illustrating a capitellar OCD in a young male without an identified cause of repetitive microtrauma, with successful arthroscopic treatment.

## 2. Case Presentation

A fifteen-year-old White male presented to our hospital's emergency room (ER) with a history of right elbow pain, oedema, and limitation of active extension after carrying heavy weights the day before. The patient also mentioned a similar episode occurring two weeks earlier, which was solved with rest and a cycle of oral nonsteroid anti-inflammatory (NSAID) drugs. The physical examination at the ER demonstrated a limited range of motion of his right elbow (10°–130°) without pronosupination restraints (80°-80°). The X-ray of right elbow showed a small bony fragment anterior to the capitellum. An ultrasound was also performed demonstrating a small intra-articular effusion with nonpure characteristics. Conservative treatment was decided, including elbow rest, local ice packs, and another cycle of oral NSAIDs.

**Figure 1:**
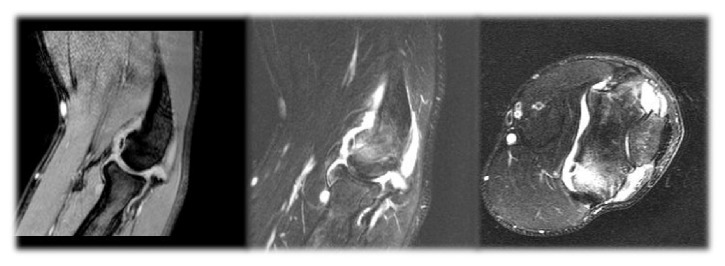
MRI of patients right elbow showing a small osteochondral capitellar lesion, intramedullary oedema, and an intra-articular loose body.

The patient remained asymptomatic for nine months when, after another episode of heavyweight bearing, the symptoms recurred. At that time, a computerized tomography (CT) scan and a magnetic resonance imaging (MRI) of the right elbow were performed showing a small osteochondral capitellar lesion, intramedullary oedema, and an intra-articular loose body (Figure 1). In this moment the patient had restricted extension (−45°) and flexion (130°) of the elbow and lateral palpation of the radiocapitellar joint elicited pain.

**Figure 2:**
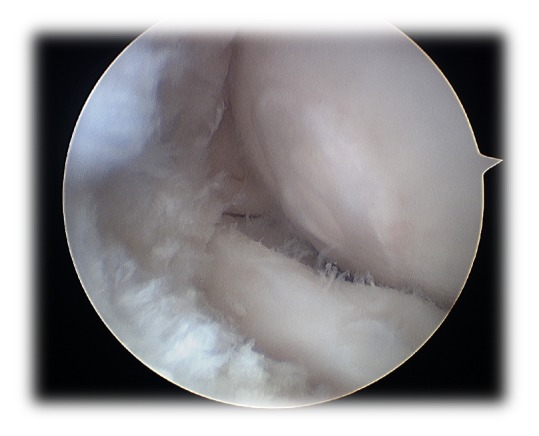
Arthroscopic finding of a type I ICRS capitellar cartilage lesion.

**Figure 3:**
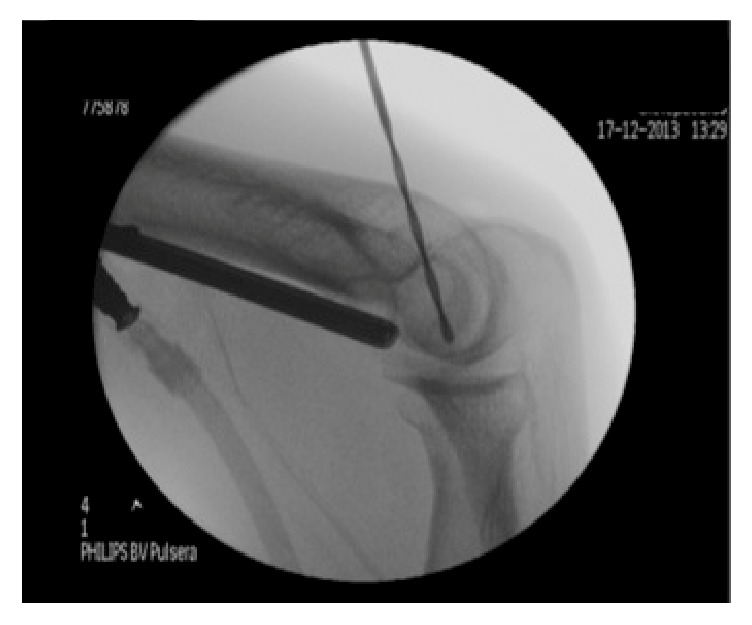
Radioscopic image of arthroscopic-assisted retrograde drilling of the capitellum.

After another episode of pain, oedema, and locking of the right elbow, occurring three months after the previous, he was proposed for arthroscopic treatment. During the arthroscopic procedure, an intact and soft capitellar cartilage (type I of International Cartilage Reconstruction Society [11]) was found, along with an associated synovitis (Figure 2). Therefore, it was decided to perform an articular synovectomy and retrograde multidirectional drilling of the capitellum (Figure 3). In the immediate postoperative period the patient had no pain or oedema, maintaining a small deficit in extension (−10°) and flexion (130°).

**Figure 4:**
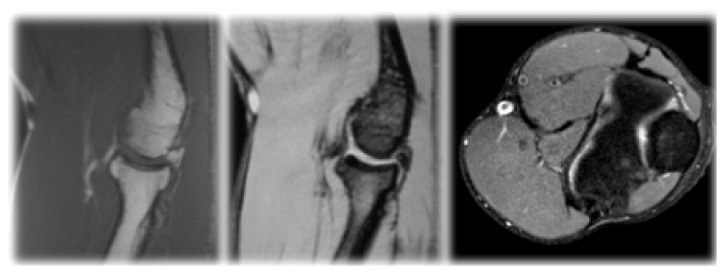
MRI of patients right elbow at one-year follow-up, demonstrating full recovery of the previously described lesions.

Actually, with a two-year follow-up the patient is still asymptomatic, without any history of recurrence of pain, oedema, or locking of the elbow. He has regained full extension (0°) and flexion (150°). There was also a recovery and normalization of the radiographic findings with the MRI showing no intramedullary oedema and no loose bodies (Figure 4).

## 3. Discussion

Our case illustrates a patient with capitellar OCD treated arthroscopically with an excellent clinical result. Regarding the demographics of the disease, this case is representative of the typical elbow OCD patient, classically described in literature: male, aged between 12 and 17 years, with an osteochondral lesion of the capitellum associated with trauma [2, 5, 12, 13]. The diagnosis was straightforward, confirmed by MRI, the imaging method of choice [2]. The fact that there was no history of repetitive microtrauma highlights the uncertainty on the pathophysiology of this disease [1–3, 5]. This finding however strengthens theories advanced by Jackson et al. [14] and Singer and Roy [15], based on the vascular vulnerability of the immature capitellum, which is supplied only by 1 or 2 end vessels entering the chondroepiphysis posteriorly. These authors hypothesized that OCD resulted from compressive insults causing vascular insufficiency to a developing chondroepiphysis.

Concerning the classification and the severity of the disease we can argue that although clinical and radiographic findings would lead us to classify this lesion as unstable according to Takahara et al. [16]—several episodes of articular locking, restriction of movement > 20°, and an intra-articular loose body—during the arthroscopic procedure no unstable lesions or loose bodies were found, leading us to reclassify this OCD lesions as stable according to the Baumgarten classifications [12] and ICRS [11].

Our strategy regarding this case is in line with the majority of papers published since the development of elbow arthroscopy techniques and follows the algorithm published by Baker III et al. [2], starting with the arthroscopy and deciding the definitive treatment based on the operative findings. In fact, as previously discussed, based on the preoperative clinical and radiographic examinations we could expect to find a fragment attached to bone or an intraarticular loose body, obliging us to consider fixation or removal/debridement and eventual microfractures. Instead we found an intact, soft cartilage and adjusted the treatment to that finding, opting by retrograde drilling, which turned out to be a wise choice. This enlightens the relevance of a first arthroscopic approach to an OCD of the capitellum.

Numerous surgical treatments for these lesions have been described [12, 13, 16]. Regarding long-term results, it was classically reported that approximately half of all affected patients would have continued elbow symptoms and develop degenerative joint disease [9, 10]. This discouraging numbers, however, represent mixed results of all the techniques described.

In recent years, there has been a trend toward early surgical management for unstable lesions with increase in arthroscopic or arthroscopy-guided surgical techniques [2, 3]. These techniques involve removal of loose bodies, drilling, debridement, abrasion chondroplasty, and fixation, with overall improvements in pain and range of motion in the short- and mid-term follow-up [16–19].

The aim of drilling is to promote a decompression that allows revascularization of the defect [20] and doing it from posterior to anterior through the humerus avoids the need for articular surface violation [3, 20]. To our knowledge this is one of the few published cases on arthroscopic treatment of capitellar OCD lesions with retrograde drilling alone. There are, however, favourable results published with retrograde drilling performed in stable osteochondral lesions of the humeral trochlea and the talus [20–23].

## 4. Conclusion

The prognosis for osteochondritis dissecans of the capitellum remains reserved.

Early identification and treatment are critical to optimizing lesion healing.

With this report the authors pretend to demonstrate a successful case in which the early arthroscopic approach was essential to the final result. Although the absence of repetitive microtrauma would not suggest it, precise diagnosis was obtained with MRI. The excellent clinical result achieved also correlated with the imaging findings, with the control MRI showing full recovery of the lesions.
